# Genetic regulation of testosterone level in overweight males from the Kazakh population and its association with hypogonadism

**DOI:** 10.25122/jml-2022-0203

**Published:** 2023-09

**Authors:** Merkhat Akkaliyev, Nurlan Aukenov, Meruyert Massabayeva, Bakytbek Apsalikov, Saule Rakhyzhanova, Muratkhan Kuderbaev

**Affiliations:** 1 Department of Surgical Disciplines, Semey Medical University, Semey, Kazakhstan; 2 Department of Health and Human Resources, Ministry of Health, Nur-Sultan, Kazakhstan; 3 Center of Scientific Research Laboratory, Semey Medical University, Semey, Kazakhstan; 4 Department of Family Medicine, Semey Medical University, Semey, Kazakhstan; 5 Department of Normal Physiology, Semey Medical University, Semey, Kazakhstan

**Keywords:** erectile dysfunction, overweight men, sex hormone binding globulin, single-nucleotide polymorphisms

## Abstract

Male hypogonadism and erectile dysfunction in different populations are associated with excess body weight. A key aspect in most studies is the metabolism of sexual hormones, primarily testosterone. At the same time, the binding protein sex hormone binding globulin (SHBG) can play a large role, as it determines the ratio of total and bioavailable testosterone in blood, i.e. both the hormone content and level of its production. Recent research has identified common mutations that affect SHBG levels, such as the rs727428 polymorphic locus, which is associated with alterations in histone protein function, affecting the regulation of ribonucleic acid (RNA) protein SHBG synthesis. Similar relationships have been observed for prevalent mutations, including rs5934505 and rs10822184, in diverse populations. This study involved 300 individuals of Kazakh nationality from the Eastern Kazakhstan region, examining three polymorphic variants of the SHBG gene (rs727428, rs5934505, and rs10822184). The participants were categorized into three groups: individuals with hypogonadism and obesity (group 1, n=85), those with excess body weight but no hypogonadism (group 2, n=70), and individuals with neither excess body weight nor hypogonadism (group 3, n=145). The frequency of mutant gene alleles impacting GPS (SHBG) synthesis in the Kazakh population was notably high, comparable to European and South-East Asian populations. However, the association between excess body weight and these mutations exhibited varying patterns. Hypogonadism was linked to decreased GPS levels, strongly correlating with total testosterone but not bioavailable testosterone. The retention of sexual functions in overweight men was not always directly related to BMI levels and GPS concentrations.

## INTRODUCTION

Testosterone concentration decreases with age as part of a natural physiological process [1]. Androgens are one of the key factors affecting the distribution of fat in a male body and are featured by the deposition of adipose tissue, mainly in the abdominal region. This can be a risk factor for developing hormonal and metabolic disorders [2]. Obesity is stated to be the main elements responsible for a decrease in general testosterone levels [3]. As urbanization continues to rise across Asia, it is expected that the forthcoming obesity epidemic will be predominantly concentrated in this region [4, 5]. Extensive research has confirmed a direct link between testosterone and obesity [6]. Testosterone deficit adversely affects many organs, and obesity causes a decrease in testosterone levels [7, 8]. Still, the duration for a decrease in androgens and clinical manifestation of hypogonadism in men can vary [9]. Physiological aging in men is determined by population-specific characteristics and individual genetic traits, including genetic mutations. Such mutations can lead to genetic polymorphism, which is predominantly present in the form of single nucleotide polymorphism (SNP). Genes are believed to play a major role in reducing androgen levels. The study considered the impact of hypogonadism on various aspects of men's physiology, including functional, biochemical, and genetic levels [10, 11]. The metabolism of sex hormones, primarily testosterone [12], is a key aspect in most studies. The regulation of testosterone production, binding to transport proteins, catabolism, and interaction with tissues is determined by the function of several key proteins. For men’s physiology, not only the synthesis of androgens is important, but also its transportation to target cells. Also, sex hormone binding protein (SHBG) can play a big role since it regulates blood correlation of total, bio-available, and free testosterone [13]. Three SNP, rs12150660, rs727428, and rs10822184, are associated with testosterone levels in populations of European ancestry [3]. However, genetic polymorphisms may vary across ethnic groups [14-18]. Particularly, the significance of the rs727428 polymorphic locus is determined by changes in the function of the marker promoters of histone proteins and increased synthesis of the ribonucleic (RNA) protein SHBG [15]. Similar relationships have been established for frequent mutations in rs5934505 and rs10822184 [19] in a number of populations. However, there is no data on SHBG polymorphism in men from Central Asia and Kazakhstan.

This study aimed to determine the relationship between mutations associated with impaired synthesis of the SHBG protein (rs727428, rs5934505, rs10822184) and hypogonadism in the Kazakh population of East Kazakhstan.

## MATERIAL AND METHODS

The study involved 300 men of Kazakh nationality living in the city of Semey, East Kazakhstan, Republic of Kazakhstan. The research adopted a case-control design, and the sample size was calculated using the EpiInfo calculator. Participants were men presenting clinical manifestations of hypogonadism who sought urologist advice.

### Inclusion criteria

- Kazakh ethnicity.
- Aged between 35 and 65 years.
- Individuals diagnosed with hypogonadism and who were actively seeking assistance from a urologist.
- Willingness to provide voluntary consent to participate in the study.

### Exclusion criteria

- Individuals aged below 35 and above 65 years.
- Patients with a diagnosis of diabetes.
- Individuals suffering from decompensated heart and/or kidney failure.
- Patients diagnosed with malignant neoplasms (cancer).
- Individuals with a documented history of smoking.
- Patients taking medications that can affect erectile function, including antihypertensive drugs, antidepressants, and hormone replacements.
- Individuals with a history of androgen replacement therapy.

After laboratory confirmation of hypogonadism, all participants provided written informed consent. Participants were divided into three groups based on their conditions:

1. Hypogonadism with excess body weight or obesity (Group 1, n=85).
2. Excess body weight without hypogonadism (Group 2, n=70).
3. No excess body weight and hypogonadism (Group 3, n=145).

Hypogonadism was diagnosed using Aging Male Screening (AMS). The evaluation criteria for the questionnaire were as follows:

- 17–26 points - no signs of testosterone deficiency;
- 27–36 points - mild signs of testosterone deficiency;
- 37–49 points - signs of testosterone deficiency of moderate severity;
- 50<points - pronounced signs of testosterone deficiency.

Body mass index (BMI) was calculated using the following formula: BMI=weight (kilograms)/height^2^ (meters). An online calculator for the Asian population was used: https://irm.kz/andrology-2/kalkulyator-indeksa-massy-tela/. Weighing was conducted with participants wearing only underwear and socks. Waist circumference was measured directly on the skin at the level of the navel in a standing position. Reference BMI values were as follows:

- normal body weight: 18.5–24.9;
- overweight: 25.0–29.9;
- 1^st^-degree obesity: 30.0–34.9;
- 2^nd^-degree obesity: 35.0–39.9;
- 3^rd^-degree obesity: 40.0 or higher.

General testosterone, sex hormone binding globulin (SHBG), and luteinizing hormone (LH) were tested on Architect i2000SR equipment (Abbott Laboratories, IL, USA) using commercial diagnostic kits (Abbott Laboratories, USA) according to the manufacturer’s instructions.

Blood samples were taken from the cubital vein between 07.00.a.m. and 10.00. a.m. on an empty stomach (after overnight fasting). Reference values for SHBG, LH, and total testosterone are 10–57 nM/L, 1.14–8.75 mIU/ml and 5.41–19.54 nM/L, respectively.

Bioactive и Free testosterone was measured by an online calculator http://www.issam.ch/freetesto.htm developed by the Hormonology Department, University Hospital of Ghent, Belgium, with inputting data for total testosterone, SHBG, and albumin.

For genetic research, we used the peripheral blood of the test subjects, collected in vacuum tubes with K2/K3 Ethylenediaminetetraacetic acid (EDTA). Isolation of genomic deoxyribonucleic acid (DNA) from blood samples was accurately performed using a commercial GeneJET Mini kit (Thermo Scientific, Vilnius, Lietuva), according to the manufacturer's instructions. The DNA concentration was assessed by Qubit 4 fluorometer (Thermo Scientific, Walthem, MA, USA). The isolated DNA was frozen and stored at -20°C.

Genotyping of 300 DNA samples, after preliminary quality and quantity checks, was carried out by real-time polymerase chain reaction (PCR) on a CFX 96 amplifier (BioRad, CA, USA) using readymade mixtures of primers and TaqMan probes, a master mix of rs754493647 gene polymorphisms, rs727428, rs5934505, and rs10822184. The total volume of 96 well plates was 25 µl, with the composition being as follows: 2x TaqMan master mix (12.50 µl), 20x master mix (1.25 µl), and DNA (11.25 µl) (20 ng) (all reagents from Life Technologies, Foster City, CA, USA). Amplification was performed for 10 min at 95°C, 50 denaturation cycles lasting 15 sec at 92°C, and annealing for 90 seconds at 60°C for all polymorphisms.

### Statistical analysis

The database was created using Microsoft Excel, and statistical analyses were conducted using SPSS version 20.0 software, licensed through Semey State Medical University, Kazakhstan. The Pearson χ2 test was employed to analyze contingency tables, while differences in continuous data sets were assessed using the Kruskal-Wallis method, following established guidelines [20]. A significance threshold of p<0.05 was applied to determine statistical significance and reject the null hypothesis.

## RESULTS

The age distribution across all groups showed no significant differences, and the average age of the groups was not significantly different (p>0.1). Among concomitant chronic somatic diseases, arterial hypertension (χ2=13.569, p=0.002) was more prevalent in Group 1 compared to Groups 2 and 3 (Table 1). The genetic test outcomes are presented in Table 2.

**Table 1:** General characteristics of groups

Index	Group 1, n=85		Group 2, n=70		Group 3, n=145		p-value
	n	%	n	%	n	%	
**Age:**							
35-40	8	9,4	9	12,9	11	7,6	0,674
41-45	14	16,5	11	15,7	25	17,2	
46-50	24	28,2	19	27,1	43	29,7	
51-55	19	22,4	17	24,3	29	20,0	
56-60	11	12,9	8	11,4	20	13,8	
61-65	9	10,6	6	8,6	17	11,7	
Average age index	48,8±5,9		50,2±5,5		50,9±6,0		0,755
**Comorbidities**							
Arterial hypertension	25	29,4	18	25,7	16	11,0	0,002
CHD (coronary heart disease)	6	7,1	4	5,7	3	2,1	0,216
Bronchial asthma, COPD	4	4,7	3	4,3	6	4,1	0,477
Renal disorders	4	4,7	2	2,9	5	3,4	0,249
GIT diseases	29	34,1	22	31,4	39	26,9	0,155
Genital pathology	6	7,1	4	5,7	9	6,2	0,307

**Table 2:** Distribution of alleles and genotypes of the transport protein gene SHBG

Allele/genotype	Group 1, n=85		Group 2, n=70		Group 3, n=145		p-value
	n	%	n	%	N	%	
**rs727428 mutation:**							
C	62	36,5	93	66,4	203	70,0	0,098
T	108	63,5	47	33,6	87	30,0	
CC	15	17,6	34	48,6	75	51,7	0,123
CT	32	37,6	25	35,7	53	36,6	
TT	38	44,7	11	15,7	17	11,7	
**rs5934505 mutation:**							
C	55	32,4	101	72,1	213	73,4	0,001
T	115	67,6	39	27,9	77	26,6	
CC	11	12,9	39	55,7	81	55,9	0,001
CT	33	38,8	23	32,9	51	35,2	
TT	41	48,2	8	11,4	13	9,0	
**rs10822184 mutation:**							
C	65	38,2	75	53,6	151	52,1	0,047
T	105	61,8	65	46,4	139	47,9	
CC	16	18,8	24	34,3	44	30,3	0,057
CT	33	38,8	27	38,6	63	43,4	
TT	36	42,4	19	27,1	38	26,2	

Differences denote statistical significance; detailed interpretation is provided in the text.

The allelic structure of the studied gene did not exhibit significant deviations from the Hardy-Weinberg distribution for any of the three mutations. When analyzing the significance of differences in allele and genotype frequencies, no significant differences were observed for the rs727428 mutation in either case (p=0.09; p=0.1). This level of difference distinguished Group 1 from Groups 2 and 3, but no difference was found between the latter groups. For the rs5934505 mutation, the corresponding values were χ2=85.179 for allele frequency and χ2=69.801 for genotype frequency (p<0.001). Less pronounced differences were identified for the rs10822184 mutation. These differences were significant in terms of the ratio of alleles (χ2=10.092, p=0.04) but not in terms of genotypes (χ2=9.184, p=0.05).

Table 3 presents the data on the distribution of participants according to the degree of excess body weight.

**Table 3:** BMI levels and distribution of participants according to the degree of excess body weight

Index	Group 1, n=85		Group 2, n=70		Group 3, n=145		p-value
	n	%	n	%	n	%	
No BMI excess	0	0	0	0	145	100	0,001
Overweight	59	69,4	50	71,4	0	0	
1^st^-degree obesity	17	20,0	15	21,4	0	0	
2^nd^-degree obesity	6	7,1	4	5,7	0	0	
3^rd^-degree obesity	3	3,5	1	1,4	0	0	
BMI, unit	31,0±2,5		30,7±2,1		22,8±1,4		0,001

Purposeful patient selection determined significant differences in the structure and average BMI between Groups 1 and 3, as well as 2 and 3. There were no differences between Groups 1 and 2 in terms of this feature, which, to a certain extent, indicates the low significance of the role of metabolic differences in the manifestation of hypogonadism between these groups.

The analysis of BMI among the study participants was conducted by assessing the distribution of patients included in the study. There was a significant difference between Groups 1–2 and 3 in terms of BMI (χ2=296.117, p<0.001). However, no significant difference in BMI was identified between Groups 1 and 2. Table 4 presents data from the hormonal status analysis.

**Table 4:** Hormonal status indicators among groups

Indicator	Group 1, n=85	Group 2, n=70	Group 3, n=145
	Q25; Me; Q75		
Testosterone total, nM/L	4.9; 6.3; 7.7	9.8; 11.9; 13.3	10.4; 12.2; 13.7
Testosterone bioactive, nM/L	3.7; 4.3; 4.7	6.5; 7.2; 8.0	5.9; 6.7; 7.8
Testosterone free, nM/L	0.12; 0.15; 0.17	0.21; 0.24; 0.28	0.19; 0.21; 0.24
SHBG, nM/L	14.1; 15.2; 16.5	20.5; 22.0; 23.3	29.8; 33.3; 37.1
LH, mIU/ml	4.1; 4.3; 4.6	3.9; 4.1; 4.5	4.0; 4.1; 4.3

Kruskal-Wallis; Q25; Me; Q75

The total, bioactive, and free testosterone content was significantly higher in both comparison groups compared to patients with hypogonadism (p<0.001; p=0.017; p=0.033). A similar pattern was identified for SHBG (p=0.012) but not for LH. The main focus was analyzing the relationship between the presence of mutations in the SHBG protein gene and the studied parameters (BMI, hormone content, and the protein itself).

The number of mutant alleles at all three loci was categorized into different groups: absence, one, two, three, or three or more. Gradation “three” was determined only in Group 3, where a greater number of mutant alleles were detected.

There was no significant dependence of BMI level on the presence and number of mutant alleles in the SHBG protein gene. However, there was a tendency for the BMI indicator to increase in individuals with a higher number of mutations.

The content of total testosterone in all groups tended to decrease both when the presence was detected and with an increase in the number of mutations. In Group 1, the dependence of total testosterone content on the number of mutations was statistically significant (p=0.033), but there was no significant dependence for bioactive testosterone. Also, no apparent features of SHBG and LH were determined to be associated with the number of identified mutations.

In Group 2, no significant relationship was observed between body weight indicators and the presence and number of SHBG protein gene mutations. The content of total testosterone in the presence of mutations was reduced and had significant differences depending on their number. The significance index in the data series was 0.023. A similar pattern was observed for the content of bioactive testosterone, with a significance level of p=0.012. At the same time, only moderate downward trends in the presence of mutations were distinctive for the SHBG level, as well as for an increase in the LH level.

In Group 3, there was no significant relationship observed between an increase in BMI and the number of mutations in the studied gene. However, similar to the previously described groups, there was a decrease in the content of total testosterone (p=0.028) and bioactive testosterone (p=0.021) depending on the number of mutations. Differences in the series of SHBG values tended to drop, and this trend became more pronounced with an increased number of mutations in the corresponding gene (p=0.033). Conversely, there were no significant differences in LH levels, similar to the other groups of patients.

## DISCUSSION

The SHBG protein is regarded as a hormone sequester crucial in controlling hormone bioavailability. A change in the concentration of SHBG can significantly affect the androgenic status of a man. In elderly and aged men, an increase in the level of SHBG in the blood is observed, which decreases as the body weight increases.

The mechanism by which the drop in SHBG levels is observed in obese patients remains debatable, but it is supposed to be the suppression of SHBG synthesis in the liver by elevated insulin concentrations [21-23].

The role of the testosterone-binding protein in the human body might be dual. On the one hand, the binding of this hormone determines a decrease in its biological activity [24, 25], while on the other hand, it specifies the preservation of its significant concentration in the deposited form [26].

In this case, the pool of bioactive testosterone is determined by the concentration associated with blood albumin and its free fraction [27]. SHBG gene polymorphism determines its differences, including discrepancies in levels or activities of key transcription factors.

Disturbances in the structure and function of the protein, as well as changes in its amount, can result in disturbances in the binding of sex hormones [28]. At the same time, there is evidence of testosterone functioning in a bound species through interaction with cellular receptors [29]. Accordingly, changes in the synthesis and structure of this protein can lead to multidirectional features of the total content and ratio of testosterone fractions [30].

A common feature is decreased SHBG protein content in the presence of mutations in the binding loci associated with the corresponding RNA synthesis [31].

The data from a number of studies has revealed that these mutations are quite common and have a unidirectional effect on the content of total testosterone. It decreases to varying degrees due to a reduction in the binding protein concentration [32]. At the same time, the amount of free testosterone, which determines the intensity of its synthesis, practically does not differ in individuals with and without these mutations [19, 33]. This phenomenon is specified by the action of hormone synthesis regulatory systems independent of the SHBG protein and adjusted by the level of free testosterone [34].

There is also evidence of a relationship between violations of testosterone levels in the blood and the development of obesity or the presence of obesity [2, 35]. Low values of the indicator in young and middle-aged men in the population have been associated with a high level of BMI [23, 36]. However, the diagnosis of hypogonadism and decreased sexual function are not always associated with obesity. There is a category of men who maintain high fertility in the presence of a moderate increase in BMI and obesity [37].

The mentioned characteristics of genetic regulation regarding testosterone binding offer a potential explanation for this phenomenon [38]. Our study found a clear relationship between the development of hypogonadism in overweight men and the content of bioactivated testosterone in the blood. Moreover, in cases of hypogonadism, this indicator was significantly lower compared to the group without elevated BMI and sexual function impairments. Conversely, these levels were even slightly higher in the group with elevated BMI but without hypogonadism.

SHBG polymorphism is a significant factor influencing testosterone concentration and can serve as an indicator for identifying men with low testosterone levels. Our data contributes to comprehending the etiology behind the early development of hypogonadism in men.

This study has some limitations, including its small sample size, which may limit the generalizability of findings. In future research, we aim to conduct a more comprehensive investigation with a larger sample to provide a more comprehensive understanding of the topic.

## CONCLUSION

The frequency of mutant alleles of genes determining the intensity of SHBG synthesis in the studied Kazakh population was high and comparable to the indicators found in the studies on European populations and Southeast Asian countries. The association between mutation rates and obesity is ambiguous. Hypogonadism was found to be caused by the decrease in SHBG level and the associated total testosterone. Testosterone associated with SHBG is not bioavailable for all target tissues, and its level depends on the concentration of SHBG, which is inversely correlated with BMI and abdominal fat. The level of the bioavailable fraction of testosterone did not depend on the concentration of SHBG polymorphic forms. This phenomenon is explained by the action of hormone synthesis regulatory systems independent of the SHBG protein and adjustable by the level of free testosterone.

## References

[1] Nieschlag E (2020). Late-onset hypogonadism: a concept comes of age. Andrology.

[2] Grossmann M (2018). Hypogonadism and male obesity: Focus on unresolved questions. Clin Endocrinol (Oxf).

[3] Tao S, Wang Z, Feng J, Hsu FC (2012). A genome-wide search for loci interacting with known prostate cancer risk-associated genetic variants. Carcinogenesis.

[4] Fui MN, Dupuis P, Grossmann M (2014). Lowered testosterone in male obesity: mechanisms, morbidity and management. Asian J Androl.

[5] Xi B, Liang Y, He T, Reilly KH (2012). Secular trends in the prevalence of general and abdominal obesity among Chinese adults, 1993-2009. Obes Rev.

[6] Fernandez CJ, Chacko EC, Pappachan JM (2019). Male Obesity-related Secondary Hypogonadism-Pathophysiology, Clinical Implications and Management. Eur Endocrinol.

[7] Yeo S, Holl K, Peñaherrera N, Wissinger U, Anstee K, Wyn R (2021). Burden of Male Hypogonadism and Major Comorbidities, and the Clinical, Economic, and Humanistic Benefits of Testosterone Therapy: A Narrative Review. Clinicoecon Outcomes Res.

[8] Fillo J, Levcikova M, Ondrusova M, Breza J, Labas P (2017). Importance of Different Grades of Abdominal Obesity on Testosterone Level, Erectile Dysfunction, and Clinical Coincidence. Am J Mens Health.

[9] Beck T, Shorter T, Brookes AJ (2020). GWAS Central: a comprehensive resource for the discovery and comparison of genotype and phenotype data from genome-wide association studies. Nucleic Acids Res.

[10] Dandona P, Dhindsa S, Ghanim H, Saad F (2021). Mechanisms underlying the metabolic actions of testosterone in humans: A narrative review. Diabetes Obes Metab.

[11] Corona G, Goulis DG, Huhtaniemi I, Zitzmann M (2020). European Academy of Andrology (EAA) guidelines on investigation, treatment and monitoring of functional hypogonadism in males: Endorsing organization: European Society of Endocrinology. Andrology.

[12] Maggi M, Filippi S, Vignozzi L, Rastrelli G (2020). Controversial aspects of testosterone in the regulation of sexual function in late-onset hypogonadism. Andrology.

[13] Avvakumov GV, Cherkasov A, Muller YA, Hammond GL (2010). Structural analyses of sex hormone-binding globulin reveal novel ligands and function. Mol Cell Endocrinol.

[14] Ohlsson C, Wallaschofski H, Lunetta KL, Stolk L (2011). Genetic determinants of serum testosterone concentrations in men. PLoS Genet.

[15] Coviello AD, Haring R, Wellons M, Vaidya D (2012). A genome-wide association meta-analysis of circulating sex hormone-binding globulin reveals multiple Loci implicated in sex steroid hormone regulation. PLoS Genet.

[16] Yassin DJ, Doros G, Hammerer PG, Yassin AA (2014). Long-term testosterone treatment in elderly men with hypogonadism and erectile dysfunction reduces obesity parameters and improves metabolic syndrome and health-related quality of life. J Sex Med.

[17] Vandenput L, Ohlsson C (2014). Genome-wide association studies on serum sex steroid levels. Mol Cell Endocrinol.

[18] Chen YP, Nie LL, Li HG, Liu TH (2016). The rs5934505 single nucleotide polymorphism (SNP) is associated with low testosterone and late-onset hypogonadism, but the rs10822184 SNP is associated with overweight and obesity in a Chinese Han population: a case-control study. Andrology.

[19] Jin G, Sun J, Kim ST, Feng J (2012). Genome-wide association study identifies a new locus JMJD1C at 10q21 that may influence serum androgen levels in men. Hum Mol Genet.

[20] Practical Statistics for Medical Research (1991). Douglas G. Altman, 1^st^ Edition. Kindle Edition.

[21] Peter A, Kantartzis K, Machann J, Schick F (2010). Relationships of circulating sex hormone-binding globulin with metabolic traits in humans. Diabetes.

[22] Jarecki P, Herman WA, Pawliczak E, Lacka K (2019). Can Low SHBG Serum Concentration Be A Good Early Marker Of Male Hypogonadism In Metabolic Syndrome?. Diabetes Metab Syndr Obes.

[23] Brand JS, Rovers MM, Yeap BB, Schneider HJ (2014). Testosterone, sex hormone-binding globulin and the metabolic syndrome in men: an individual participant data meta-analysis of observational studies. PLoS One.

[24] Keevil BG, Adaway J (2019). Assessment of free testosterone concentration. J Steroid Biochem Mol Biol.

[25] Simó R, Sáez-López C, Barbosa-Desongles A, Hernández C, Selva DM (2015). Novel insights in SHBG regulation and clinical implications. Trends Endocrinol Metab.

[26] Morgentaler A, Traish A, Hackett G, Jones TH, Ramasamy R (2019). Diagnosis and Treatment of Testosterone Deficiency: Updated Recommendations From the Lisbon 2018 International Consultation for Sexual Medicine. Sex Med Rev.

[27] García-Cruz E, Alcaraz A (2020). Testosterone deficiency syndrome: Diagnosis and treatment. Actas Urol Esp (Engl Ed).

[28] Wan Q, Xie Y, Zhou Y, Shen X (2021). Research progress on the relationship between sex hormone-binding globulin and male reproductive system diseases. Andrologia.

[29] Goldštajn MŠ, Toljan K, Grgić F, Jurković I, Baldani DP (2016). Sex Hormone Binding Globulin (SHBG) as a Marker of Clinical Disorders. Coll Antropol.

[30] Salas-Huetos A, Maghsoumi-Norouzabad L, James ER, Carrell DT (2021). Male adiposity, sperm parameters and reproductive hormones: An updated systematic review and collaborative meta-analysis. Obes Rev.

[31] Ozzola G (2016). Essay of sex hormone binding protein in internal medicine: a brief review. Clin Ter.

[32] Thaler MA, Seifert-Klauss V, Luppa PB (2015). The biomarker sex hormone-binding globulin-from established applications to emerging trends in clinical medicine. Best Pract Res Clin Endocrinol Metab.

[33] Aydın B, Winters SJ (2016). Sex Hormone-Binding Globulin in Children and Adolescents. J Clin Res Pediatr Endocrinol.

[34] Harada N (2018). Role of androgens in energy metabolism affecting on body composition, metabolic syndrome, type 2 diabetes, cardiovascular disease, and longevity: lessons from a meta-analysis and rodent studies. Biosci Biotechnol Biochem.

[35] Pivonello R, Menafra D, Riccio E, Garifalos F (2019). Metabolic Disorders and Male Hypogonadotropic Hypogonadism. Front Endocrinol (Lausanne).

[36] Basualto-Alarcón C, Llanos P, García-Rivas G, Troncoso MF (2021). Classic and Novel Sex Hormone Binding Globulin Effects on the Cardiovascular System in Men. Int J Endocrinol.

[37] Vandewalle S, De Schepper J, Kaufman JM (2015). Androgens and obesity in male adolescents. Curr Opin Endocrinol Diabetes Obes.

[38] Fui MN, Dupuis P, Grossmann M (2014). Lowered testosterone in male obesity: mechanisms, morbidity and management. Asian J Androl.

